# Double fossilization in eukaryotic microorganisms from Lower Cretaceous amber

**DOI:** 10.1186/1741-7007-7-9

**Published:** 2009-02-20

**Authors:** Ana Martín-González, Jacek Wierzchos, Juan-Carlos Gutiérrez, Jesús Alonso, Carmen Ascaso

**Affiliations:** 1 Departamento de Microbiología-III, Facultad de Biología, Universidad Complutense, 28040-Madrid, Spain; 2 Centro de Ciencias Medioambientales, CSIC, Serrano 115 bis, 28006-Madrid, Spain; 3 Museo de Ciencias Naturales de Álava, c/Siervas de Jesús 24, Victoria-Gasteiz, Spain

## Abstract

**Background:**

Microfossils are not only useful for elucidating biological macro- and microevolution but also the biogeochemical history of our planet. Pyritization is the most important and extensive mode of preservation of animals and especially of plants. Entrapping in amber, a fossilized resin, is considered an alternative mode of biological preservation. For the first time, the internal organization of 114-million-year-old microfossils entrapped in Lower Cretaceous amber is described and analyzed, using adapted scanning electron microscopy in backscattered electron mode in association with energy dispersive X-ray spectroscopy microanalysis. Double fossilization of several protists included in diverse taxonomical groups and some vegetal debris is described and analyzed.

**Results:**

In protists without an exoskeleton or shell (ciliates, naked amoebae, flagellates), determinate structures, including the nuclei, surface envelopes (cortex or cytoplasmic membrane) and hyaloplasm are the main sites of pyritization. In protists with a biomineralized skeleton (diatoms), silicon was replaced by pyrite. Permineralization was the main mode of pyritization. Framboidal, subhedral and microcrystalline are the predominant pyrite textures detected in the cells. Abundant pyritized vegetal debris have also been found inside the amber nuggets and the surrounding sediments. This vegetal debris usually contained numerous pyrite framboids and very densely packed polycrystalline pyrite formations infilled with different elements of the secondary xylem.

**Conclusion:**

Embedding in amber and pyritization are not always alternative modes of biological preservation during geological times, but double fossilization is possible under certain environmental conditions. Pyritization in protists shows a quite different pattern with regard to plants, due to the different composition and cellular architecture in these microorganisms and organisms. Anaerobic sulphate-reducing bacteria could play a crucial role in this microbial fossilization.

## Background

A major problem for understanding the origin of life, microbial evolution and phylogeny is the lack of microbial fossils. It is especially evident when we consider the available well-preserved record of pluricellular organisms, animals and plants [1]. Microfossils are not only useful for elucidating biological macro- and microevolution but also the biogeochemical history of our planet. Amber is a fossilized resin originating from the trunk and roots of certain trees, particularly of the genera *Agathis *and *Hymenaea. *It acts as a natural embedding agent and it has properties similar to amorphous polymeric glass [2]. Amber consists of a complex mixture of terpenoid and/or phenolic compounds. The organisms that are embedded in it are maintained in their three-dimensional form and their morphological features are preserved, making it possible to compare them with their present-day descendants [3]. Trapping in amber is not the most frequent mechanism of preservation of biological systems, especially of the so-called soft-bodied fossils (for example, nematodes and insects); mineralization as a result of both microbial and abiotic processes is the most common mechanism. Two main mechanisms of fossilization by mineralization are recognized: permineralization, which is the result of early infiltration and permeation of cells and/or tissues by mineral-charged water; and replication of morphology in authigenic minerals which are mainly a product of bacterial activity [4]. Mineralization in pyrite is called pyritization. Pyrite is the most common sulphide mineral found in marine argillaceous sedimentary rocks, where it can occur in a variety of crystallographic and textural forms [5]. Pyritization is considered an important mode of preservation and/or fossilization in animals and plants, with or without a skeleton or cuticle [6-8]. Although little detailed work has been published on pyrite in fossils, three grades of biological preservation by pyritization have been recognized [5,9]: 1) permineralization, involving pyrite precipitation in cellular cavities or cell walls made of poorly biodegradable components such as cellulose and chitin; 2) formation of mineral coats, which is usually involved in the preservation of very degradable biological components. These pyrite coats have a limited and clearly defined thickness; and 3) formation of mineral casts or moulds. This style of preservation causes the greatest degree of biological information loss, since only the fossil outline is preserved. The main difference between these three modes of preservation by pyritization is the extent of mineral precipitation.

One important feature in the pyritization process is pyrite texture. The term texture is used to define the characteristics (size, shape, crystallinity) and contact relationships among pyrite crystals. The external form and shape of any aggregate of pyrite crystals is referred to as morphology [5]. Pyrite textures have been used to determine the mechanism of pyritization [7] and even (considering pyrite morphologies, also) the paleoenvironmental and paleo-redox status [10]. However, the process of pyritization is still poorly understood and many questions remain unanswered; for instance, if it is a selective process, how do the internal tissues or cell interior become pyritized? [7]. Thus, in general, pyritization and trapping in amber are two different taphonomic modes of biological fossilization. Only a few specimens of completely pyritized insects in amber or with a coat of pyrite between the insect carcass and the surrounding amber have been reported, as mentioned in the excellent review on insect taphonomy by Martínez-Delclòs et al [11]. Pyrite has also been found in the spores of a lichen-like inclusion in Eocene amber [12]. In these cases, the mode of formation is unknown, but according to some authors, pyrite may penetrate by microfractures in the amber and connect with the organisms, precipitating [13,14]. Moreover, some microcrystals of marcasite (a mineral with the same chemical composition as pyrite that crystallizes in a different crystal system) were detected in the interior of some insects from French Cenomanian amber [15].

Paleomicrobiological studies on amber are very scarce. The most ancient eukaryotic microorganisms found in amber date back to the Triassic (220 My ago) [16]. In addition, there are some observations, most of them by optical microscopy, on the microbiocoenosis in German [17-20], French [21] and American [22-24] ambers from the Middle and Upper Cretaceous. However, there is no data on the potential mechanism (or mechanisms) involved in microbial fossilization. To date, we have not found any information about the cytoplasmic organization of these microfossils or any other relevant ultrastructural data. For some years, we have been analyzing and identifying eukaryotic microoganisms trapped in the Lower Cretaceous amber from Peñacerrada (Álava, Spain), considered exceptional from both a geological and paleontological point of view. It is one of the highly fossiliferous amber deposits from the Lower Cretaceous, relatively rare in relation with Upper Cretaceous resins [11,25]. Preliminary studies [26,27] indicate that amber deposits from Peñacerrada contain diverse and abundant communities of prokaryotic and especially eukaryotic microorganisms. Our previous observations by optical microscopy of diverse fossilized protists suggested the existence of a remarkable morphological stasis [28]. In the present paper, we have studied in detail, by SEM-BSE (scanning electron microscopy in backscattered electron mode) associated to EDS (energy dispersive X-ray spectrometry) microanalysis, the degree of biological conservation and the mechanism/s involved in the preservation of the eukaryotic microorganisms embedded in this ancient amber. The results obtained provide relevant evidence which contradicts some previous paleobiological theories about biomineralization, in particular pyritization. The importance of this information in paleo- and geomicrobiology is discussed.

## Results

In the amber site at Peñacerrada (Peñacerrada II in previous references, [25]) the most productive source of amber nuggets was associated with centimetre-sized nodules of pyrite with 2 mm octagonal crystals. Microcrystalline pyrite also was detected as irregular crusts over amber lumps and in the core of lignite fragments [25]. Amber deposits were always associated with the coal layers that are more abundant in the middle part of the formation [29,30].

**Figure 1:**
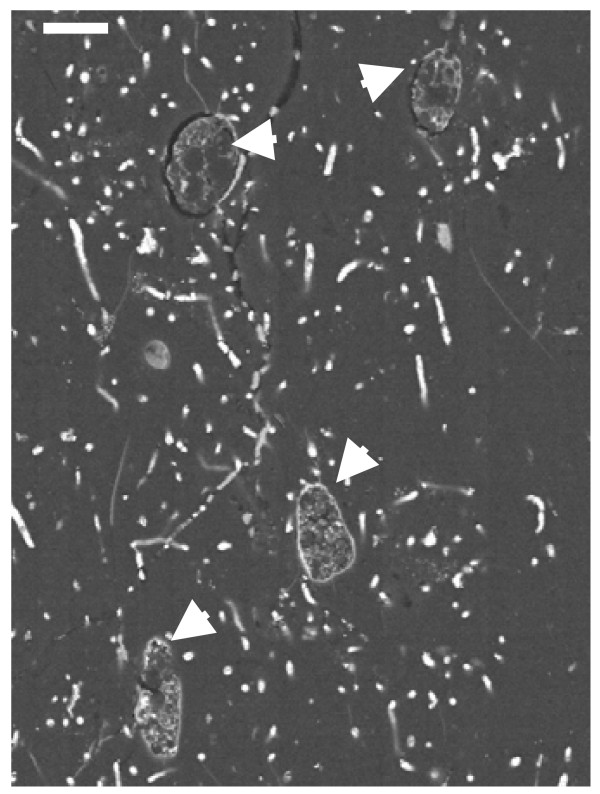
**General view of a transverse section of Lower Cretaceous amber from Peñacerrada, showing entrapped protists (arrows) and some fragments of fungal hyphae**. Scale bar 20 μm.

The pattern of pyrite deposition and crystallization presented differences according to each protist morphotype, that is, the cellular organization and composition. However, the general pattern of pyrite crystal distribution was similar in all protists. The size of the cells may have been an important factor in the pyrite texture. Figure 1 illustrates the general appearance of different pyritized microorganisms in amber, including different protists and fragments of fungal hyphae. Small naked amoebae are quite frequent in the Cretaceous amber studied.

**Figure 2:**
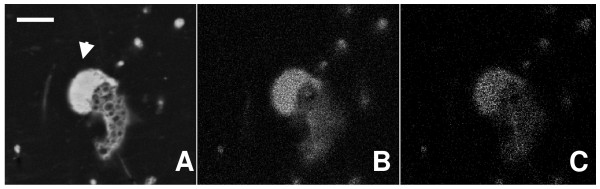
**General view of a monopodial pyritized amoeba**. (A). S and Fe content microanalysis integrated image in a monopodial fossil amoeba, similar to the extant genus *Hartmannella*. Arrow points to pyritized hyaloplasm of the leading pseudopodium. (B) Content and distribution of S. (C) Content and distribution of Fe. Scale bar 10 μm.

Figure 2A shows the SEM-BSE image of a small pyritized monopodial naked amoeba, very similar to the extant genus *Hartmannella*. The sulphur and iron contents, detected by microanalysis, are illustrated respectively in Figure 2B and 2C; the quantity of sulphur seems to be almost double the iron content, which corresponds to their proportion in pyrite (FeS_2_). As may be observed, the cytoplasm contained numerous vacuoles or vesicles and the pyrite deposition is more intense in the hyaloplasm, a jellified hyaline cap located at the front of the leading pseudopodium [31]. However, the texture of pyrite is always microcrystalline in all cellular locations.

**Figure 3:**
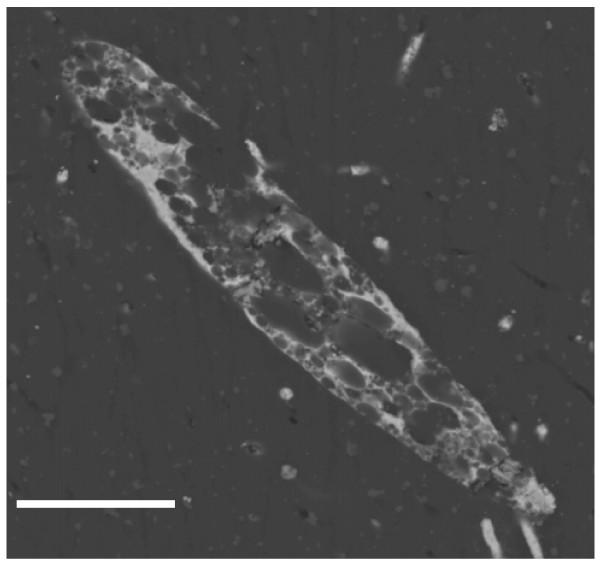
**Cytoplasmic view of a pyritized flagellate, similar to the extant genus *Euglena*.** Note the presence of numerous vesicles and vacuoles, and the microcrystalline deposits in surface envelopes. Scale bar 20 μm.

**Figure 4:**
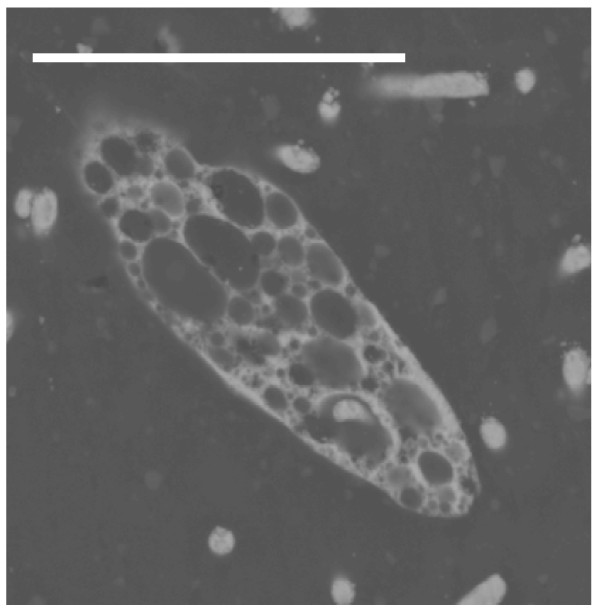
**Trabecular distribution of pyrite microcrystals in a small flagellate**. Scale bar 20 μm.

**Figure 5:**
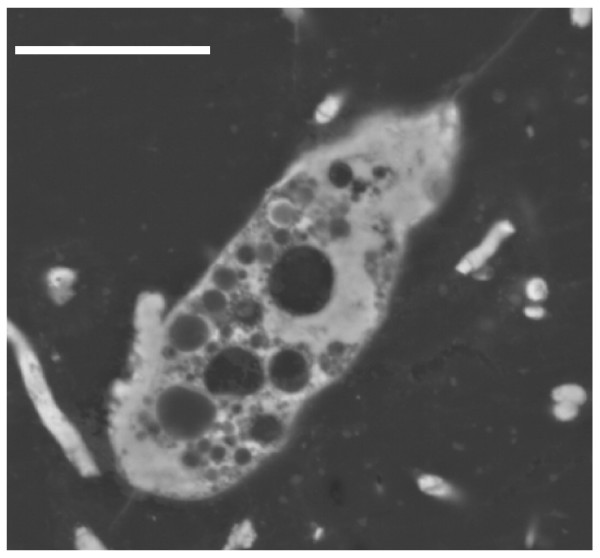
**Lateral view of a small heterotrophic flagellate, similar to the extant genus *Chilomonas*.** Observe that cytoplasm is infilled by massive polycrystalline pyrite. Scale bar 10 μm.

Both heterotrophic and photosynthetic flagellates of different taxa have been identified in amber. In all of them the pyrite microcrystalline deposits are more intense inside the plasma membrane and cell wall. The cytoplasm of these pyritized cells presents a trabecular appearance with many vesicles and vacuoles of different sizes delimited by a microcrystalline network (Figures 3 and 4). In some cases, the cytoplasm is partially infilled with massive polycrystalline pyrite (Figure 5).

**Figure 6:**
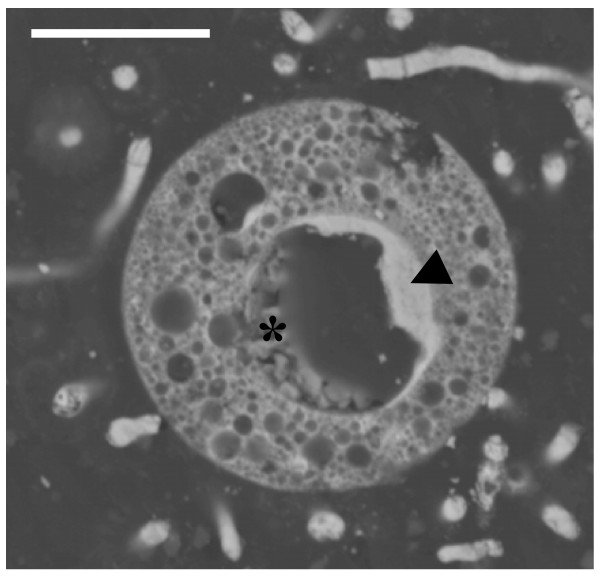
**Cross section of a small flagellate, similar to the extant photosynthetic genus *Chlamydomonas*, showing the trabecular organization of pyrite microcrystals in the cytoplasm**. The arrow points to the pyritized nuclear envelope. The asterisk is on the subhedral bigger crystals that partially infilled the nuclear core. Scale bar 10 μm.

When the cellular section prepared for SEM-BSE includes the nucleus, it is possible to observe that the pyritization pattern of this structure is very different. Figure 6 shows the cross section of a pyritized small cell, which probably corresponds to the photosynthetic extant genus *Chlamydomonas*. In this case, the nuclear envelope has been replaced by a layer of discrete spheroidal or cubo-octahedral microcrystals or grains (<0.1 μm), densely packaged. Equimorphic organization of pyrite microcrystals was similar to that from pyrite framboids. However, framboids usually occur in clusters of finite size. The organic core of the nucleus (nucleoplasm) has been degraded and is partially infilled by densely packed groups of subhedral crystals that are bigger (<1 μm, >0.1 μm) than those from the biomineralized nuclear envelope (Figure 6).

**Figure 7:**
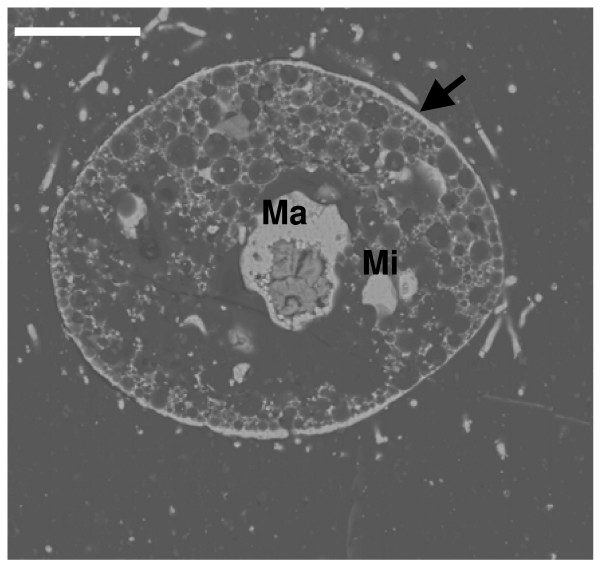
**Longitudinal section of a pyritized ciliate**. The cortex (Arrow) and the nuclei; macronucleus (Ma) and micronucleus (Mi) are the most mineralized cellular structures. Scale bar 20 μm.

**Figure 8:**
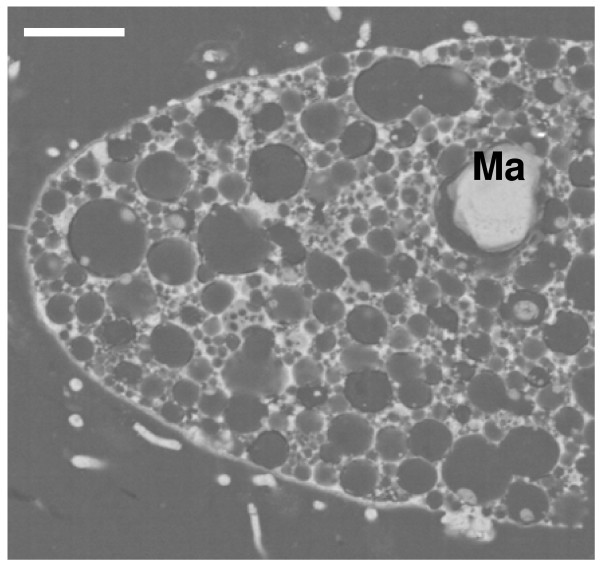
**Detail of a fossilized ciliate similar to the extant genus *Paramecium***. Note the numerous vesicles that probably contained H_2_S during early pyritization. Ma: pyritized macronucleus. Scale bar 20 μm.

The pyritization pattern in ciliated protozoa is similar to that in flagellates. In these protists, there are two morphological and functional types of nuclei; the vegetative macronucleus and the germinative micronucleus. Subhedral or cubo-octahedral crystals of pyrite preferentially infilled the nuclei. Disorganized aggregates of regular microcrystals with a framboid-like appearance infilled the space corresponding to the double nuclear membrane. The size of framboids depended on the size of the nuclei (macronucleus/micronucleus) of each species (Figures 7 and 8). Ciliated protozoa have no cell wall, but they present a complex structure around the cytoplasm, called a cortex, that includes cilia, kinetosomes and fibrillar-associated structures and two or three membranes. In all micrographs, the cortex is infilled by pyrite microcrystals forming a layer without uniform width (Figures 7 and 8).

**Figure 9:**
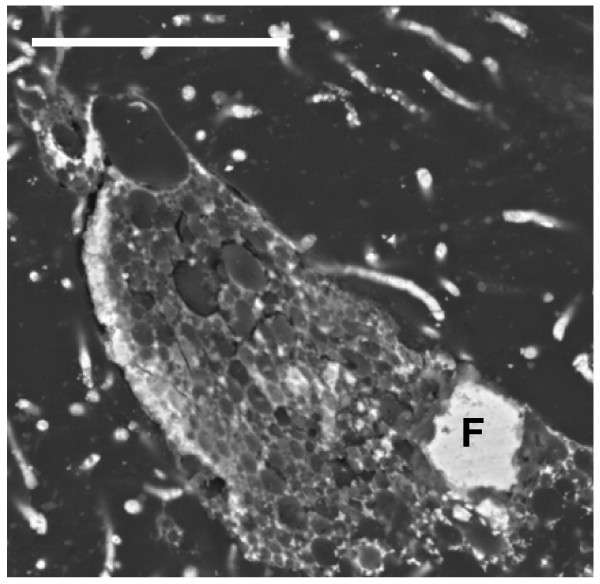
**Longitudinal section of a pyritized diatom entrapped in amber, similar to the extant genus *Cymbella***. Note the framboid (F) in the nucleus. The Si of the skeleton has been changed by pyrite. Scale bar 20 μm.

Pyritization also affected those protists with a mineralized external skeleton. Figure 9 shows the longitudinal section of a diatom similar to the extant genus *Cymbella*. The species of this genus have a frustule made of very pure silica, coated with a layer of organic material. It is usually easy to identify them, because the valves are asymmetrical around the apical axis and the cells present a slight dorsoventrality. The microanalysis associated with the SEM-BSE indicates that replacement of Si by pyrite took place in these microfossils. The cytoplasm appears very vacuolated with a complex network of microcrystals. A framboid-like pyrite can be observed in the location of the nucleus.

**Figure 10:**
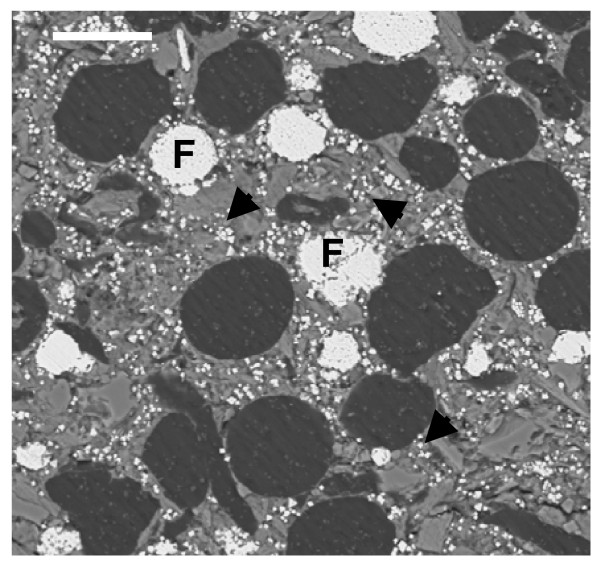
**General appearance of a pyritized plant debris**. Note the pyrite framboids (F) and the octahedral crystal (arrows) distributed inside the parenchyma cells surrounding the vascular elements. Scale bar 25 μm.

**Figure 11:**
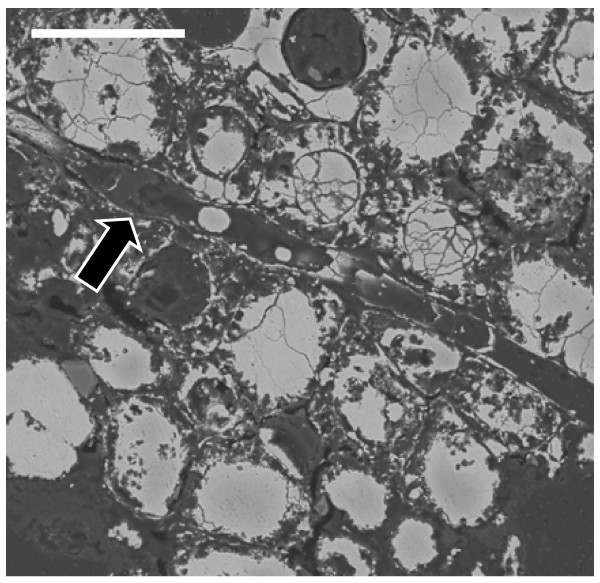
**General view of secondary xylem vessels infilled by micropolycrystalline pyrite.** Arrow points to an empty tracheid. Scale bar 50 μm.

**Figure 12:**
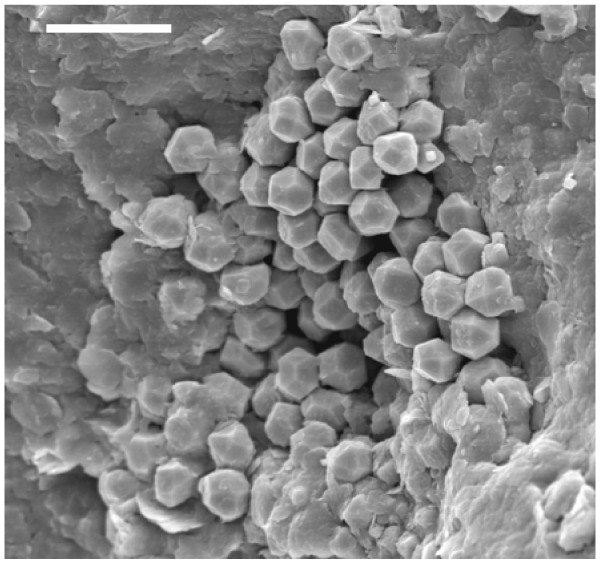
**Detail of pyrite crystals located in the sediments surrounding the amber nuggets**. Note the octahedral and subhedral cubo-octahedral crystalline formations. Scale bar 25 μm.

**Figure 13:**
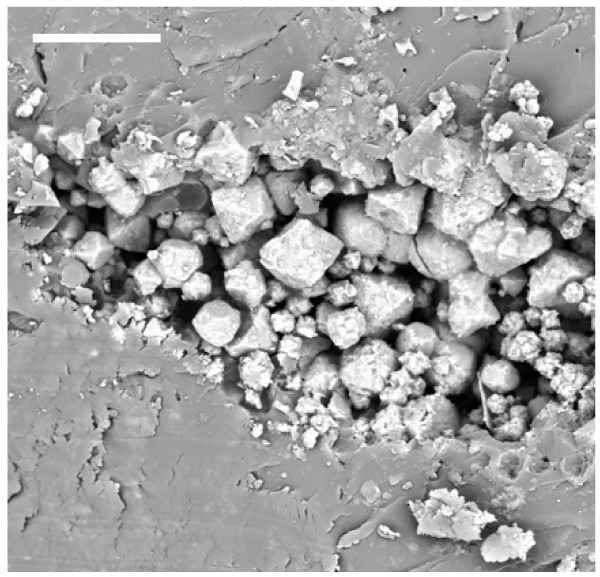
**Detail of a plant debris embedded in amber showing a large crystalline formation of pyrite**. Scale bar 20 μm.

**Figure 14:**
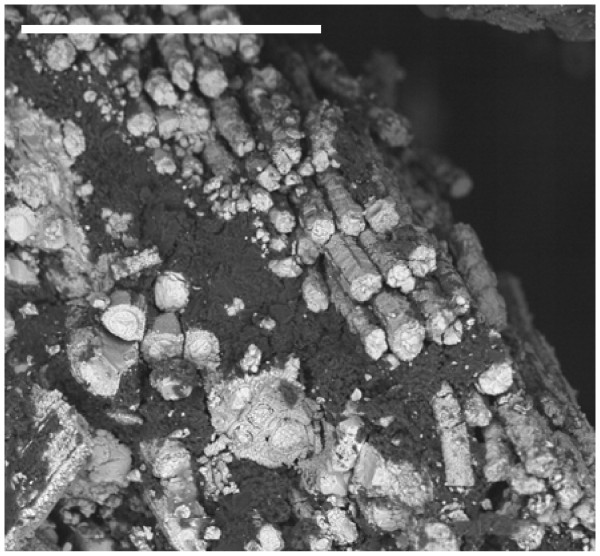
**Pyritized plant debris entrapped in the sediments located close to the amber nuggets.** Observe that the xylema vessels are densely infilled by pyrite crystals. Scale bar 50 μm.

**Figure 15:**
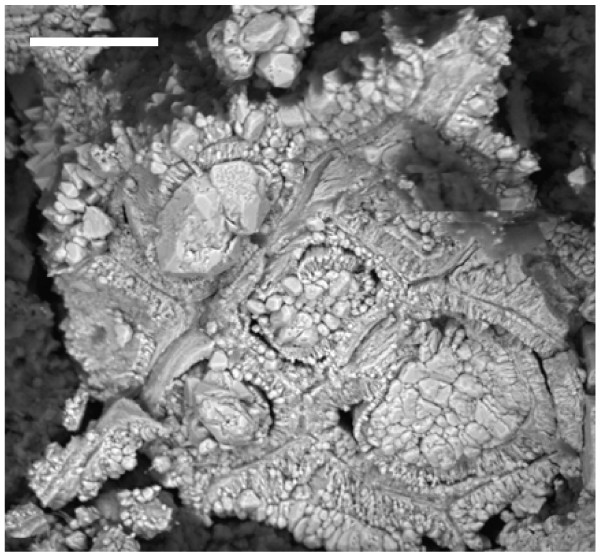
**Detail of a zone from Figure 14 showing, with higher augmentation, the distribution of pyrite crystals and microcrystals**. Scale bar 20 μm.

**Figure 16:**
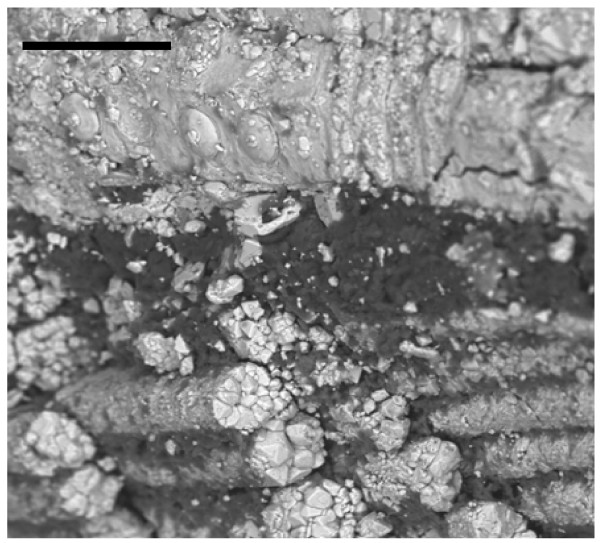
**Pyritized vegetal debris located very close to but outside the amber nuggets showing the parenchyma cells infilled by microcrystalline pyrite**. Scale bar 50 μm.

In the same amber nuggets, we also found abundant pyritized vegetal microdebris, usually containing numerous pyrite framboids with variable sizes (<10 to 50 μm) (Figure 10). In Figure 11, we can observe very densely packed polycrystalline pyrite formations that infill different elements of the secondary xylem. It may be observed that tracheids are not infilled by pyrite. We also processed some samples of the sediments next to the amber deposits. Numerous crusts of pyrite with different textures, mainly octahedral and subhedral cubo-octahedral, were detected in the sediments (Figure 12). These large crusts of pyrite crystals also occurred embedded in the amber, but located outside the cells (Figure 13). Pyritized vegetal debris can be observed more clearly when not entrapped in amber, making it possible to analyze the different textures of pyrite. The lumina of xylem vessels appeared infilled by densely packed subhedral or cubo-octahedral pyrite (Figure 14). The texture associated with the inner surface of cell walls is microcrystalline (Figure 15). We did not detect framboids associated with the xylem vessels, only with parenchyma. However, parenchymal cells more frequently appear completely infilled with microcrystalline pyrite (Figure 16).

## Discussion

All previous studies of the pyritization of soft parts have analyzed vegetal and animal fossils. Our paleomicrobiological analysis provides new information, and a substantial part of these data do not support some postulates and general affirmations about pyritization. In all observed and analyzed microbial fossils, permineralization of cells seemed to be the main mode of pyritization. It is stated that in a permineralized tissue, pyrite occurs inside the boundary between the organism and the surrounding sediment [5]. In (macro)fossils, preservation by this mechanism usually involves the replacement by pyrite of more degradable components, such as the cell walls of plants. The poorly biodegradable tissues, such as cellulose or chitin, may be preserved by the precipitation of pyrite in their pore spaces or infilling of cellular cavities [32]. However, the pyritization of labile tissues (for example, skin and muscle fibres) has not been documented [4,33]. Experimental induction of pyritization in microalgae resulted in the infilling of *Chlorella *with iron sulphides and a limited disruption of cells [34]. This is the first time that it has been possible to analyze the pyritization pattern of a unicellular organism, including the internal architecture. The protist cortical structures, irrespective of their organization (cortex, plasmatic membrane, cell wall) and composition, are important centres of pyritization and they appear infilled by microcrystals of pyrite. This does not correspond to a mineral coat, since the pyrite does not occur in a thin and well-defined zone, immediately outside the organism/cellular boundary. Another cellular structure that is preferentially replaced by pyrite is the nucleus or nuclei, despite being composed of easily biodegradable macromolecules (DNA and proteins). It is remarkable that the pyrite texture of the nuclear envelope was different to that of the nucleoplasm. This pyritization pattern is different from those reported in plants, where the pyrite is precipitated in fluid-filled spaces in the plant, from cell-sized voids to spaces between fibrils in the cell wall [7,34,35]. Furthermore, there is no evidence for iron sulphides penetrating the cells during experimental pyritization [34].

Different cell wall composition in these two types of biological systems, as well as the absence of this structure in some protists (for example, ciliates), might explain the different patterns observed in protists and plants. When the protist has a skeleton, such as *Cymbella*, the siliceous frustule is replaced by a pyritized skeleton made of microcrystals. Dispersive X-ray analysis has shown the complete replacement of silica by pyrite. However, we cannot observe the degree of ornamentation preservation because we have only obtained longitudinal sections. Numerous assemblages of marine pyritized diatoms from the Tertiary (Palaeocene-Eocene) have been described [36-38]. Two different modes of pyritization have been reported; a change of the siliceous frustule by a well-preserved pyritized skeleton and the crystallization of pyrite in internal cavities of the diatoms, preserving only the external morphology.

The most frequent pyrite textures found in our fossilized protists were microcrystalline and framboidal. However, outside cells but inside the amber and also in the sediments surrounding the amber deposits, we found mainly octahedral or subhedral pyrite in clusters of different sizes. In studies of fossil plants from the Eocene London Clay, Grimes et al reported different textures in adjacent cells of the same vegetal tissue. They stated that there is no exclusive relationship between cell type and a particular texture or combination of textures [7]. This fact has been interpreted as evidence of the development of isolated chemical micro-environments and the progressive changes in pore water chemistry as a result of microbial activity and reactant availability during burial [7]. Our observations of pyritized plant debris from inside Cretaceous amber nuggets and the adjacent sediments showed some differences. We found no framboidal pyrite within the lumen of xylem vessels, a common occurrence in plant fossils from London Clay. On the contrary, the texture found in the lumina of xylem vessels is subhedral. In secondary xylem, microcrystalline pyrite infilled the lignified cell walls.

These results do not agree with the four-stage model proposed to explain the pyritization of fossils from London Clay [7], which assumed two affirmations which are very difficult to support from a microbiological point of view. First, it was proposed that pyrite nucleation may have been facilitated by the availability of oxidizing agents or bacterial biofilms. Sulphate-reducing bacteria, the microorganisms involved in biological formation of pyrite, are obligate anaerobes. Microbial biofilms favour the existence of anaerobic environmental conditions that are incompatible with the presence of oxidizing agents. Second, the existence of distinct pyrite textures in two adjacent cells is explained as a consequence of microhabitats [7]. The existence of microhabitats in biofilms and microbial mats, in which very different physiological groups of bacteria are distributed according to their physiological requirements, is well known. However, it is difficult to assume that there are diverse chemical micro-environments in the same eukaryotic microbial cell. The pyritized cell in Figure 6 is only around 12 μm in diameter and it showed at least two different pyrite textures.

The amber nuggets from Peñacerrada had frequent inclusions of vegetal debris, including root fragments, suggesting the microbial and vegetal materials were included in the resin in the soil, before their transport. The sedimentology of the amber deposits, including the type of mineralization found in the layer which was the most productive source of amber lumps, is representative of the early diagenesis caused by diverse populations of anaerobic bacteria that result from the deposition of organic matter, which impedes oxygen diffusion across the sediment [39]. The formation and accumulation of pyrite during the early diagenesis is controlled by a number of environmental factors, including availability of iron, sulphate and organic carbon, anoxic conditions, pyrite oxidation and hydrodynamics [10]. These factors are crucial for the optimal growth and metabolism of sulphate-reducing bacteria, the main microbial group involved in biological pyritization. With regard to the taxonomic aspects of the amber deposit, according to Alonso et al [25], amber lumps from nearby forests were transported in suspension by rivers and deposited in low-energy areas as floodplains and swamps. In this concentration process, well-preserved lumps and altered ones come together.

It is important to assign a temporal sequence to both pyritization and trapping in amber fossilization processes that protists underwent more than 114 My ago. Microanalysis of sediments adjacent to the amber deposits indicated that plant debris were pyritized, so the pyritization seems to be a prior and independent process to cellular inclusion into the resin. However, the pyritization may have continued for some time after the cells were embedded in the resin. Two reasons support this hypothesis. Organic matter degradation (decay) and mineralization (pyritization) represent two competing forces during early fossilization, and the amount of detail preserved in a biological fossil is usually a reflection of the end result of the relative timing of these two polar forces [1,6]. Trapping in amber preserves tissues and cells because it arrests organic degradation; however, mineralization is also occurring. It is therefore possible to observe some details of cellular morphology of the protists in amber. Secondly, chemical analysis from Peñacerrada amber demonstrated anoxic conditions inside fossilized resin [40].

At present, microbial involvement in both pyrite precipitation to form sediments and pyritization (mineralization) of organisms, tissues and cells, is the subject of intense controversy. Authors favouring a chemical origin support their hypothesis with two main facts; pyrite framboids have been synthesized in some laboratories (see [41]), and microfossils of bacteria (in sediments, amber and so on) are relatively rare, exhibiting very low cellular concentrations although bacterial concentrations are, at present, usually high in many environments [42,43]. However, more evidence supports early diagenetic pyrite forms, natural pyrite framboids and biological pyritization being a result of microbial processes, although some abiotic chemical reactions might also be involved. First, successful experimental synthesis of framboids has been achieved in only a few instances in which high temperature (150 to 300°C) and the addition of SO_2 _were employed, or by increasing the redox potential of the systems [41]. Second, fossilized bacteria with distinct morphologies have been observed in pyrite-containing sediments and oncoids by SEM and transmission electron microscopy [42-45]. Third, the results from very diverse experimental pyritization assays with plants show that anoxic sediments or cultures of sulphate-reducing bacteria are needed, that oxidizing conditions inhibits pyritization and that biomineralization of plants cells is rapid (24 to 80 days), which contributes to cell preservation [8,34,46]. Finally, it must be added that the chief physiological group of bacteria that lead pyritization, the sulphate reducers, appears to be a very ancient and diverse group of microorganisms [47]. Moreover, there is isotopic evidence of microbial sulphate reduction more than 2.7 Gy ago, in the early Archaean era [48].

Additional valuable information is provided by the characteristics of sediments located around the amber deposits where pyritized (micro/macro) fossils appeared. In the detailed description of fossiliferous amber deposits in New Jersey (Upper Cretaceous) by Grimaldi et al [49], where two pyritized insects were reported, the amber was buried in sediments that were highly reducing, as revealed by strong sulphurous gases during excavations, and by the abundance of pyrite and marcasite. A similar reducing environment, with H_2_S emission, fragments of carbonized microcrystalline wood (lignite) and octagonal crystals of pyrite crusts has been described in Peñacerrada sediments [25]. This type of mineralization is representative of deposition and degradation of organic matter by anaerobic bacteria [39]. For all of the above reasons, we think that the anaerobic sulphate-respiring bacteria play a key role in the biomineralization (pyritization) of Cretaceous protists and other microorganisms found in amber from Peñacerrada.

## Conclusion

Ultrastructural examination and microanalysis of fossilized protists in Peñacerrada's Lower Cretaceous amber deposits support the view that, under certain environmental conditions, it is possible to find biological records which underwent two different mechanisms of fossilization – pyritization and entrapping in amber. Therefore, pyritization and embedding in resin are not always alternative modes of biological preservation. These uncommon double fossilizations indicate that anaerobic conditions and abundant organic matter as well as available and permanent/semi-permanent freshwater were present in the ecosystem of Peñacerrada during Lower Cretaceous, previous to the biological inclusions in the resins. The comparative study of fossilized plant debris inside amber nuggets and those in sediments surrounding these nuggets showed that, at least in this case, inclusion in resin was a process prior to permineralization. In protists, the pattern of pyritization showed some differences from those observed in vegetal debris, probably due to the drastic differences of composition and cellular organization in these two biological kingdoms.

## Methods

### Collection and processing of amber and sediments

Several tons of deep amber-containing sediments were extracted. Amber deposits were then separated from the rocks, pyrite nodules, lignite fragments and sandstones using a standard concrete mixer. Amber has a low specific gravity and generally floats or is buoyant in water. The concrete mixer was fed with a continuous flow of water, and the slurry resulting from mixing with rocks was poured through a 5 mm mesh. Solids of the lowest density (that is, amber and lignite) were collected from the surface, while dense sediments settled to the bottom of the concrete mixer. Each extraction process lasted 10 or 15 minutes. Regardless of size, amber lumps were the first to come out onto the sieve, followed by denser lignite fragments. Material deposited at the bottom of the concrete mixer consisted mainly of sand, fragments of rock, and pyrite nodules, which was free of amber, revealing the efficiency of this method in processing large quantities of amberiferous rocks.

Each piece of amber was carefully examined under a stereoscopic microscope to detect biological inclusions, such as small arthropods and plant debris. All amber pieces containing biological inclusions were embedded in the epoxy resin Epotek 301 to eliminate the mirror effect of internal cracks and to avoid natural oxidation and darkening. Internal zones of deeper sediments surrounding amber pieces were collected manually and stored aseptically after processing for electron microscopy.

### SEM-BSE with EDS microanalysis examination

Small pieces of amber were embedded in epoxy resin and after polymerization each block was cut and finely polished [26]. The polished surfaces were carbon coated and observed using DSM 940 A Zeiss and DSM 960 A Zeiss microscopes, equipped with a four-diode semiconductor BSE detector and a Link ISIS microanalytical EDS system. SEM-BSE and EDS examinations of the samples were performed simultaneously. Operating conditions are specified elsewhere [50]. The use of BSE in SEM yields high-magnification images with contrast attributable to differences in average atomic number of the target [51]. It has been applied successfully in the examination of pyritized plant material [35].

## Abbreviations

EDS: energy dispersive X-ray spectroscopy; Gy: 1000 millions of years; My: millions of years; SEM: scanning electron microscopy; SEM-BSE: scanning electron microscopy in backscattered electron mode.

## Authors' contributions

AMG and JCG undertook analysis and microbial identification. JW, JA and CA provided and processed samples. JW and CA carried out the microscopy techniques. AMG, JCG, JW, JA, CA conceived and wrote the paper. All authors have read and approved the final manuscript.
